# Do Couple-Based Interventions Make a Difference for Couples Affected by Cancer?: A Systematic Review

**DOI:** 10.1186/1471-2407-12-279

**Published:** 2012-07-06

**Authors:** Tim W Regan, Sylvie D Lambert, Afaf Girgis, Brian Kelly, Karen Kayser, Jane Turner

**Affiliations:** 1 Centre for Translational Neuroscience and Mental Health, Faculty of Health, School of Medicine and Public Health, The University of Newcastle, Newcastle, Australia; 2 Translational Cancer Research Unit, Ingham Institute for Applied Medical Research, South Western Sydney Clinical School, Faculty of Medicine, The University of New South Wales, Sydney, Australia; 3 Kent School of Social Work, University of Louisville, Louisville, KY, USA; 4 School of Medicine, University of Queensland, Brisbane, Queensland, Australia

## Abstract

**Background:**

With the growing recognition that patients and partners react to a cancer diagnosis as an interdependent system and increasing evidence that psychosocial interventions can be beneficial to both patients and partners, there has been a recent increase in the attention given to interventions that target couples. The aim of this systematic review was to identify existing couple-based interventions for patients with cancer and their partners and explore the efficacy of these interventions (including whether there is added value to target the couple versus individuals), the content and delivery of couple-based interventions, and to identify the key elements of couple-based interventions that promote improvement in adjustment to cancer diagnosis.

**Method:**

A systematic review of the cancer literature was performed to identify experimental and quasi-experimental couple-based interventions published between 1990 and 2011. To be considered for this review, studies had to test the efficacy of a psychosocial intervention for couples affected by cancer. Studies were excluded if they were published in a language other than English or French, focused on pharmacological, exercise, or dietary components combined with psychosocial components, or did not assess the impact of the intervention on psychological distress (e.g., depression, anxiety) or quality of life. Data were extracted using a standardised data collection form, and were analysed independently by three reviewers.

**Results:**

Of the 709 articles screened, 23 were included in this review. Couple-based interventions were most efficacious in improving couple communication, psychological distress, and relationship functioning. Interventions had a limited impact on physical distress and social adjustment. Most interventions focused on improving communication and increasing understanding of the cancer diagnosis within couples. Interventions were most often delivered by masters-level nurses or clinical psychologists. Although most were delivered in person, few were telephone-based. No difference in efficacy was noted based on mode of delivery. Factors associated with uptake and completion included symptom severity, available time and willingness to travel.

**Conclusion:**

Given effect sizes of couple-based interventions are similar to those reported in recent meta-analyses of patient-only and caregiver-only interventions (~*d*=.35-.45), it appears couple-based interventions for patients with cancer and their partners may be at least as efficacious as patient-only and caregiver-only interventions. Despite evidence that couple-based interventions enhance psycho-social adjustment for both patients and partners, these interventions have not yet been widely adopted. Although more work is needed to facilitate translation to routine practice, evidence reviewed is promising in reducing distress and improving coping and adjustment to a cancer diagnosis or to cancer symptoms.

## Background

Cancer is the leading cause of disease-related burden in Australia and accounts for nearly one-fifth of the total disease burden [1]. In 2010, approximately 115,000 Australians were diagnosed with cancer, with 1 in 2 men and 1 in 3 women diagnosed before the age of 85 [1]. As the number of people living beyond initial diagnosis is increasing, so is the time during which the disease sequelae and psychosocial consequences must be managed. The ability of patients and their partners^a^ to manage cancer challenges has been shown to impact on their short and long-term adjustment to the disease [2]. Therefore, reducing psychological distress, increasing coping ability, and improving the quality of life (QoL) of individuals with cancer and their partners or caregivers are priorities for researchers and clinicians [3,4].

### Conceptualisations of adjustment to cancer

One of the most popular frameworks for understanding adjustment to life stressors, such as cancer, was described by Lazarus & Folkman in their seminal book “Stress, Appraisal, and Coping” [5]. Lazarus & Folkman described coping with stressful events as an active process, placing emphasis on the context in which stressors are presented and must be dealt with. It follows that how individuals cope with a cancer diagnosis is partially determined by their ongoing appraisal of new and unfamiliar challenges that arise during the course of their illness. Findings from a recent meta-analysis of appraisal and coping styles [6] found that when cancer is appraised as a threat, individuals tend to engage more in problem-focused coping, whereas when cancer is appraised as a harm/loss or as a challenge, emotion-based coping is more likely to be used. Meaning-making coping was later added to the framework as a means of reconciling an appraisal of a stressor that is incongruent with one’s personal beliefs and goals [7]. There is an intuitive clarity within Lazarus and Folkman’s approach to coping, but translation into clinical practice has not been so straightforward. Coyne and Racioppo [8] highlighted that understanding coping and coping styles has not necessarily improved researchers’ understanding of the efficacy of psychosocial interventions aimed at increasing coping. They suggested that despite numerous reviews and meta-analyses of coping interventions, the lack of consistency in intervention types, experimental designs, outcome measures, and a lack of methodological rigour make it difficult to enunciate the key elements of these interventions that can produce improvements in individuals’ and partners’ psychological distress.

### Current evidence about interventions to promote adjustment

Several meta-analyses have been conducted in recent years to better understand the impact of psychosocial interventions for people with cancer [9-14]. For instance, Meyer and Mark [9] conducted a meta-analysis of controlled studies that implemented various psychosocial interventions for individuals with different cancer types and found these to have small effects on outcomes measured such as emotional adjustment, functional adjustment, treatment and disease symptoms (*d*=.17 - .28). However, when controlling for intervention type, non-behavioural interventions (e.g., interventions that focus on increasing social support and allow for expression of emotion) showed stronger effects on emotional adjustment measures (*d*=.39) in comparison to cognitive-behavioural or psycho-education interventions. Another meta-analysis by Sheard and Maguire (1999) attempted to minimise heterogeneity of outcome measures by focusing on psychological interventions for anxiety and depression, conducting separate analyses for each of these outcomes. Overall, a moderate effect of psychological interventions on anxiety (*d=*.42) was found. Larger effects on anxiety and depression were found for studies where treatments included more than eight hours of therapy, and were conducted with a more experienced therapist [12]. Longer-term interventions (minimum 12 weeks) had a greater impact on QoL than short-term interventions (*d*=1.19, *d*=.47) [11]. This suggests that at least some of the effects of these psychosocial interventions may be attributed to the relationships formed between individuals and their therapist or therapeutic group.

### Psycho-educational interventions

Group psycho-education interventions (i.e., interventions where the primary aim was to educate patients on the management of psychological symptoms) were found to have significantly stronger effects on anxiety (*d=*1.59, *p*<.01) than group therapy that excluded psycho-education (*d*=.27) [12]. A similar trend was found on depression outcomes, as group psycho-education was more efficacious (*d*=.94) than group therapy that excluded psycho-education (*d*=.42) [12]. Consistent with this, individual format interventions were less efficacious than group format therapies (relaxation only *d=*.03; individual therapy; *d*=.30). This strong, positive effect of psycho-education was also found on measures of QoL in a separate meta-analysis conducted by Rehse and Pukrop [11].

### Cognitive behavioural therapy -based interventions

A meta-analysis by Osborne, Demoncada, and Feuerstein [10] compared psycho-education interventions with Cognitive Behavioural Therapy (CBT) interventions on measures of anxiety and depression. No evidence was found to support the efficacy of psycho-education interventions, due largely to the small number of studies included. CBT interventions, on the other hand, were shown to have strong effects on both anxiety and depression (*g=*1.99, *g*=1.21, respectively). Further, Osborne et al. found evidence to suggest that individual-based CBT interventions were more efficacious than group-based CBT interventions. A similar result was found in Tatrow and Montgomery’s [13] meta-analysis of CBT techniques for improving distress in individuals with cancer. CBT delivered in an individual format (*d*=.48) produced larger effect sizes than therapy delivered in a group format (*d*=−.06). A meta-analysis of the moderators of effects in psychosocial interventions for breast cancer patients found that individual interventions may result in greater effect sizes than group-based interventions; however, this difference was eliminated when controlling for interventions that included homogenous cancer types versus interventions that included heterogeneous cancer types [14].

### Interventions directed towards partners

Research has traditionally focused on the impact of cancer on patients, and only recently has significant attention been paid to the impact of a diagnosis on partners [15]. Patients and partners often describe similar reactions to a cancer diagnosis, including shock [16-18], distress [19-21], anxiety [16,17,20], depression [16,17,20], fear and uncertainty [17,18,22], and denial [17]. Moreover, there is evidence to suggest that partner or caregiver anxiety may be associated with patient anxiety, and may influence other illness adjustment outcomes including depression, fatigue, and symptom management [23]. Thus, interventions that address the concerns of partners are essential. Recent reviews and meta-analyses suggest that interventions targeting caregivers (who are often the partners of patients with cancer) can significantly improve coping ability, QoL, communication, sexual functioning, and self-efficacy, and can significantly reduce caregiver burden [24-29]. More specifically, a meta-analysis by Northouse et al. [28] found that caregiver interventions were superior to usual care in reducing anxiety (*g*=.20) and improving physical functioning (*g*=.22 - .26) and family and marriage relationships (*g*=.20). Moderator analysis revealed that a greater number of intervention sessions (*M=*5.2 sessions) and a greater number of intervention hours (*M*=7 h) had a positive influence on coping ability. This is similar to the findings from the aforementioned meta-analysis by Sheard and Maguire and Rehse and Pukrop [11,12]. Harding and Higginson [26] presented evidence to suggest that the mode of delivery for caregiver interventions is also an important consideration. Although caregivers in these studies found both individual and group formats acceptable, some formats were preferred for selected content. For instance, the content of individual format interventions targeted problem solving skills, emotional expression, and pain management education [26]. The content of group-based, caregiver-only interventions was generally similar to group-based patient-only interventions, with a focus on information exchange, shared experiences, and the promotion of self-help [26]. Somewhat surprisingly, few interventions have focused on improving the partners ability to provide physical assistance to patients, beyond the management of pain [24]. Two reviews [25,27] have highlighted that despite some success, a lack of methodological rigour, and the heterogeneity of research design, theoretical frameworks and outcome measures limits the generalisability of caregiver-only interventions.

Despite evidence of the substantial impact of a cancer diagnosis on both patients and partners [15] and interventions targeting patients and partners separately having at least a moderate impact on coping and adjustment to the disease, there have been few studies investigating the efficacy of couple-based coping interventions [30]. McLean and Jones found some evidence to support to use of a couple-based intervention for palliative care patients, though cited a lack of studies to make concrete recommendations [31]. A review of couple-based interventions by Baik and Adams included 14 studies, and concluded that couple-based interventions can lead to improvements in dyadic-level adjustment [32]. Although they provided an overview of the results of each intervention, this review did not provide particular depth with regard to intervention efficacy. Moreover, the authors also included studies that did not report partner outcomes [3,33], and one case study [34]. Although these can still be considered couple-based interventions, the lack of partner outcomes limits interpretations of the differential effects of a couple-based intervention for patients and partners. Hopkinson and colleagues undertook a review of couple-based interventions and their impact on symptom management and other health behaviours [35]. They concluded that couple-based interventions can improve adjustment to cancer, and provided a concise overview of the studies that relieved symptoms psychosocial distress. However, the authors provided little detail with regard to the size of the differences between intervention and control couples, or the specific measures used to assess the various psychosocial domains. Hopkinson and colleagues also included studies that did not report partner outcomes, and in some cases did not require a partner to be involved in the intervention [36,37]. Finally, Scott and Kayser recently undertook a review of couple-based interventions to improve sexuality and body image for women with cancer [38]. They found that some interventions that included partners produced greater effect sizes than interventions that focused on patients only. Moreover, intervention effects tended to be maintained for longer following a couple-based intervention compared to a patient-only intervention. These improvements and their maintenance are hypothesized to be based on improvements in dyadic coping and increased knowledge of the patient’s diagnosis and treatments.

There is growing evidence demonstrating the efficacy of patient-only and caregiver/partner-only interventions in reducing psychological distress and improving QoL, and longer-term improvements in sexual functioning and body image when partners actively participate in interventions [9-13,25-28,38]. This review will complement existing reviews by examining 1) the efficacy of couple-based interventions across a wide range of outcome measures, 2) how the content of specific couple-based interventions is tailored and delivered to couples and 3) which elements of couple-based interventions seem most promising in reducing psychological distress and improving adjustment among patients and partners.

## Method

A systematic review was undertaken to explore the efficacy, content, and delivery of couple-based interventions. The heterogeneity of intervention content, intervention delivery, cancer type, outcome measures, intervention length, and follow-up length made the implementation of a meta-analysis unfeasible [39,40]. To maximise methodological quality, this review was conducted in accordance with the guidelines suggested by the Preferred Reporting Items for Systematic Reviews and Meta-analyses (PRISMA) [41].

### Inclusion criteria

Studies were included if they:

· Evaluated a psychosocial intervention (psychological, behavioural, or educational) for people with cancer AND their partners.

· Used an experimental or quasi-experimental design

· Were published between January 1990 and May 2010. [NB: This period was chosen as the majority of couple-based interventions began to emerge around this time.]

· Targeted individuals diagnosed with cancer (any type and any stage along the illness trajectory).

· Were published in English or French [languages spoken by the authors].

· Included depression, anxiety, distress, or QoL as an outcome measure.

Studies were excluded if:

· They compared interventions that were relatively similar in their focus.

· Pharmacological, exercise, or dietary elements were the central component of the intervention, with psychosocial elements being secondary (in order to delineate the efficacy of the psychosocial component).

· They focused specifically on sexuality and sexual functioning as outcome measures. While related to psychological distress, these were deemed to be a separate consideration worthy of more specific attention.

· They did not report patient and partner outcomes separately.

### Literature search

CINAHL, PSYCINFO, MEDLINE, EMBASE, and ISI Web of Science were searched for relevant articles. The key search terms were (couple* OR partner* OR support* OR caregiver* OR carer* OR family OR spouse* OR husband* OR wife OR wives OR close relative(s), OR next of kin(s), significant other(s), OR couple(s), OR family, OR families, OR relative(s) AND (cancer OR neoplasm*).

The NOT command was used to exclude the following terms: Nutrition OR physical activity OR diet OR child* OR youth OR adolescent*. Reference lists of recent literature reviews, unpublished articles, doctoral theses, and of all individual articles retrieved were also searched.

In addition, individual researchers known to do work in this area were contacted by e-mail and asked if they had studies currently under review or in-press that may be eligible for inclusion in the review.

### Data extraction

The titles and abstracts for all identified papers were assessed for relevance by the first author and were rejected if the study did not meet the inclusion criteria. The abstracts of the remaining studies were then assessed against the inclusion and exclusion criteria by two reviewers and those that met the criteria were retained for full review. Where there was disagreement between reviewers, consensus was reached through discussion. Two reviewers independently extracted data using a standard data collection form, which included authors, country, aims, sample size, cancer type, intervention components, intervention duration, and method of delivery of the intervention.

### Methodological criteria

The methodological quality of the studies included in this review was assessed using the criteria described by the Effective Public Health Practice Project (EPHPP) [42] and the National Health and Medical Research Centre (NHMRC) guidelines for hierarchically assessing levels of evidence [43]. The EPHPP is a reliable, valid and comprehensive tool for use in detecting bias within intervention studies, and is considered suitable to be used in systematic reviews of the effectiveness of interventions [44,45]. Six domains from each study were given a rating from 1- ‘strong’ to 3 - ‘weak’. These domains were: selection bias, study design, confounders, blinding, data collection methods, and withdrawals and dropouts. Each study was then given a global rating from 1- ‘strong’ to 3 - ‘weak’. Studies were rated as strong if they: 1) included participants likely to represent the target population, 2) used an randomised controlled trial or controlled clinical trial (CCT) design, 3) controlled for confounders, 4) blinded participants to the research question, and blinded outcomes assessors to participant status, 5) reported reliability and validity of the measures used, or used outcomes measures with known reliability and validity, and 6) reported an attrition rate of 20% or less. The methodological quality of each study was assessed by three authors. Uncertainty regarding the quality of any studies was resolved through discussion among three of the authors. Using the guidelines described in the EPHPP [42], eight studies had a global rating of ‘strong’ [23,46-52], whereas the remaining 15 studies were rated as ‘moderate’. The most common reason for a study not receiving a rating of ‘strong’ was due to a low response rate from eligible participants, which led to otherwise ‘strong’ articles being rated as ‘moderate’.

The National Health and Medical Research Centre (NHMRC) has published guidelines for hierarchically assessing levels of evidence to indicate the degree to which bias has been minimised [43]. A level of II reflects evidence obtained from an appropriately designed randomised controlled trial, level III-1 reflects evidence from a pseudo-randomised controlled trial, and level III-2 reflects evidence obtained from comparative non-randomised studies with concurrent controls (e.g., cohort studies). Six studies in this review had an evidence level of II [46,47,49,50,53,54], twelve had an evidence level of III-1 [4,23,48,51,52,55-61], and five had an evidence level of III-2 [62-66].

## Results

**Figure 1:**
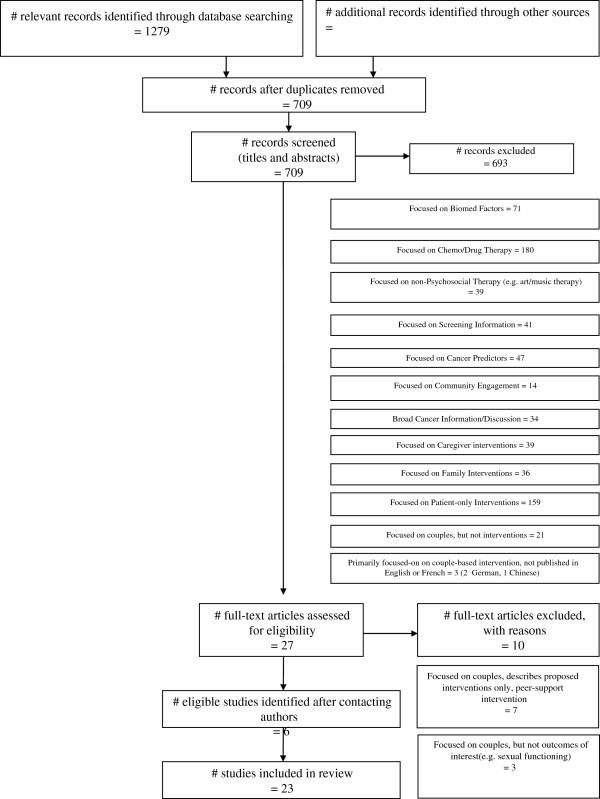
Flowchart of literature search.

Twenty-three couple-based intervention studies were included in this review. Initially 1279 articles were identified across the electronic databases: 237 articles identified from the CINAHL database, 144 from PSYCINFO, 406 from MEDLINE and 492 from ISI Web of Science. Of these, 570 duplicates were removed. The titles and abstracts of the remaining 709 studies were screened, and 27 studies were retained for full-text review. Details of the excluded studies and entire literature search are presented in Figure 1. Of the 27 studies kept for full review, 10 were subsequently excluded: seven described a planned couple-based intervention (no efficacy data available) or described a peer support intervention (e.g., support from a cancer survivor, not necessarily known to the patient); and three because the primary focus was not relevant to this review (e.g., were not specifically psychosocial interventions). Six additional studies were identified after making contact with researchers in the field.

Additional file 1: Table S1 outlines the key information for each of the studies included in this review. This includes the author, design, methodological quality rating [42], evidence level rating [43], description of the intervention and control conditions, including length of intervention and delivery format, sample, the outcomes measured in each study, and significant outcomes with effect sizes (Cohen’s *d*[67]). Along with the content of each intervention, each intervention was classified as either a dyadic intervention (where both patient and partner engage in the intervention simultaneously and with similar roles), a coaching intervention (where the partner takes on more of a ‘proxy’ therapist role), or an individual intervention (where patients and partner receive the same or similar intervention separately). Effect size*)* was calculated using the difference in mean scores between the intervention and control group (for patients and partners separately) at specified time-points, divided by the pooled sample standard deviation [68]. All effect sizes are shown as positive values, regardless of the measure used, to indicate improvement for the experimental group compared to the particular comparison group.

### Characteristics of couple-based interventions

#### Classification of interventions

The couple-based interventions reviewed here can be classified under three broad categories. Thirteen studies [23,46,47,49,50,58,60-66] had a primary focus on improving communication between partners. Eight [4,51-54,56,57,59] studies had a primary focus on developing specific skills to enhance coping ability (e.g., relaxation techniques, stress management, obtaining information). Two studies [48,55] had a primary focus on educating patients and partners on specific elements of cancer and cancer care (e.g., symptom management, potential treatment side-effects).

#### Delivery of interventions

Additional file 1: Table S1 outlines the details of the delivery of these couple-based interventions. Fifteen interventions were delivered to couples face-to-face [4,47,49-54,58,60,61,63-66], four were delivered by telephone [23,46,56,62], three were delivered face-to-face and over the telephone [48,57,59], and one was delivered using face-to-face, over the telephone and through educational videos [55]. Twenty of the interventions were delivered to both patients and partners at the same time [4,47-54,56-66]. Three of these interventions were classified as ‘coaching’ interventions [48,50,59], as they tended to utilise the partner as a proxy therapist, whose role in the intervention was to assist the patient. The remaining 17 interventions were classified as ‘dyadic’ interventions, as they addressed the couple as unit. Three interventions were delivered to couples separately (via telephone, though with related content) [23,46,55]. Seven interventions were delivered by psychologists or clinical psychology doctoral students [4,47,49,56,58,62,64], one was delivered by PhD level psychologists and licensed clinical social workers [65], three were delivered by masters-level therapists from psychology or social work backgrounds [50,53,60], six were delivered by masters-level nurses [23,48,51,52,54,57], one was delivered by a psychiatric nurse counsellor [23], two were delivered by nurses (experience and qualifications not otherwise described) [55,59], and three were delivered by health care professionals described as therapists or counsellors (experience or professional background not provided) [61,63,66]. Seven of the interventions required those delivering them to undergo specific training of intervention protocols, ranging in length from six-hours to a four-day seminar [50-53,55,56,62]. All studies followed a specific intervention protocol, and all but seven studies [48,49,58,61,63,64,66] outlined their plans to maintain treatment fidelity.

#### Characteristics of participants in couples-based interventions

Table 1 presents a summary of participant characteristics. The majority of patients was Caucasian, highly educated, diagnosed with breast or prostate cancer, and aged in their early 50s. There was an even proportion of patients across disease stages.

**Table 1:** Description of patients and partners

	**Patients**	**Partners**
**Mean, SD, Age (years)**	54.48 (6.12)	53.37 (4.34)
**Gender, % female**	73.71	55.77
**Ethnicity, %**		
*Caucasian*	81.90	84.98
*African-American*	10.15	5.34
*Hispanic*	2.92	2.79
*Asian*	1.33	1.40
*Other*	3.71	5.25
**Cancer Site, %**		
*Breast*	48.67	
*Prostate*	22.76	
*Gynaecological*	1.95	
*Lung*	9.83	
*Head & Neck*	0.45	
*Leukaemia*	1.20	
*Non-Hodgkins*	0.15	
*Hodgkins*	0.11	
*Gastrointestinal*	10.93	
*Brain*	0.30	
*Other*	3.64	
**Cancer Stage, %**		
*Stage I*	27.72	
*Stage II*	25.91	
*Stage III*	21.18	
*Stage IV*	25.19	
**Partner Relationship, %**		
*Spouse*	85.85	
*Family Member*	10.32	
*Friend*	3.83	
**Education, %**		
*High School or less*	31.23	36.43
*Some university/University graduate*	68.77	63.57
*Some university/University graduate*	68.77	63.57

#### Participant uptake and attrition

Based on 17 studies that provided clear eligibility and randomisation data, 2315 out of 4631 (49.99%) eligible couples were randomised into a couple-based intervention (*M*=136.18, *SD*=119.07). Across all studies, 84.49% of patients (*N*=1956, *M*=115.06, *SD*=100.60) and 82.55% of partners (*N*=1911, *M*=112.41, *SD*=100.64) who were randomised into an intervention provided data at the first follow-up point. At the final follow-up point, 73.19% of patients (*N*=1678, *M*=111.87, *SD*=76.16) and 71.30% of partners (*N*=1607, *M*=107.13, *SD*=74.72) provided data. The most common reasons for withdrawal were the distance to the intervention being too far, burden of illness too great, or participants felt that the intervention did not meet their needs. Furthermore, some studies suggested participants were more likely to drop out if they had late-stage cancer, lung cancer, poor physical functioning at baseline, or poor emotional connection or warmth to their partner at baseline [4,58,59], though none of the studies reported any pattern of systematic attrition.

### Outcomes

Outcome measures used for patients and partners are shown in Additional file 1: Table S1.

#### Quality of life (QoL)

QoL was conceptualised as the couples’ perceptions of how cancer has impacted everyday aspects of their physical and emotional functioning and activities. Among the five studies that assessed global QoL, four used self-report measures [51-54] and one used clinician ratings [50]. One study reported weak to moderate improvements in QoL for patients immediately following the intervention [52] whereas the remaining four studies reported no significant change immediately following the intervention. Four studies assessed change between six and 12 months post-intervention and only one of these reported improvements at follow-up compared to the control group [53]. Four assessed partners’ QoL [51-54], and two studies [52,54] reported weak to moderate improvements immediately following the intervention, and also at six, and 12 month follow-ups.

#### Psychological distress

Across studies psychological distress was conceptualised as emotional distress, anxiety, depression, worry, negative thoughts, and/or negative mood. Of the 18 studies that assessed psychological distress [4,23,46-50,54,55,57-64,66], nine reported greater improvements for intervention patients than control patients [4,23,46-48,50,55,58,59], and three studies reported improvements compared to baseline scores [63-65] immediately following the intervention. Six studies reported greater improvements for intervention patients than control patients [4,46-48,50,58], and one study [64] reported great scores compared to baseline at the final follow-up point. For partners, two studies reported significant improvements for intervention groups compared to control groups immediately following the intervention [46,47], and three studies reported within-group improvements compared to baseline scores [63-65]. Four studies reported improvements for intervention partners compared to control group partners at the final follow-up point [46-48,58], and one study reported within-group improvement at the final follow-up point compared to baseline [64].

#### Physical distress

Physical distress was conceptualised as the impact cancer and treatments (including side effects) had on individuals’ physical functioning, pain, and fatigue. Of the eight studies that assessed physical distress specifically (i.e., separate to global QoL measures) [46,47,51,52,55,56,59,61], three reported greater improvements for intervention patients than control patients immediately following the intervention [46,56,59]. Three studies reported greater improvements for intervention patients than control patients at the final follow-up point [46,55,59]. Two studies assessed partners’ ratings of their own physical distress [46,55], and one study assessed partner’s ratings of how much they were affected by the patient’s physical distress [52]. In one study, intervention partners also reported less physical symptoms (of their own) compared to control partners immediately following the intervention [55]. Another study reported that intervention partners reported being significantly less affected by the patient’s physical distress compared to control partners immediately following the intervention [52].

#### Sexuality

Sexuality was conceptualised as the sexual functioning and satisfaction of patients and partners since their diagnosis. Of the five studies that assessed sexuality [4,47,48,52,56], one study reported greater improvements for intervention patients than control immediately following the intervention [4]. Two studies reported greater improvements for intervention patients than control patients at the final follow-up point [4,48]. For partners, one study reported improvements immediately following the intervention and at the final follow-up [48].

#### Social adjustment

Social adjustment was conceptualised as the ability of patients and partners to maintain family, vocational, and social roles. Six studies assessed social adjustment for both patients and partners [46,53,55,59,65]. Three studies found greater improvements for intervention patients compared to control patients immediately following the intervention [46,53,59]. Two studies found greater improvements for intervention patients compared to control patients at the final follow-up point [46,53]. Two studies reported greater improvements for intervention partners compared to control partners immediately following the intervention [46,59], and one study found greater improvements for intervention partners than control partners at the final follow-up point [46].

#### Relationship functioning

Relationship functioning was conceptualised as the quality of the relationship between patients and partners and their satisfaction with the relationship. Of the nine studies that assessed relationship functioning [47-49,58,60,61,63,64,66], five reported greater improvements for intervention patients compared to control patients immediately following the intervention [47-49,58,60,63]. Four studies greater improvements for intervention patients compared to control patients at the final follow-up point [47,48,58,64]. Four studies reported greater improvements for intervention partners compared to control partners immediately following the intervention [47-49,58], and four studies greater improvements for intervention partners compared to control partners at the final follow-up point [47,48,58,64].

#### Appraisal variables

Appraisal was conceptualised as how patients and partners perceived and understand their abilities and their emotional status. Three studies assessed illness appraisal, caregiving appraisal, hopelessness appraisal, and uncertainty appraisal [51,52,54]. One study [51] reported that intervention patients had less negative illness appraisal than control patients at the final follow-up point, whereas partners had less negative illness appraisal immediately following the intervention. The same study [51] also reported less hopelessness appraisal for intervention patients than control patients at immediately following the intervention, and at the final follow-up point. Another study by the same team [52] reported an improvement in hopelessness appraisal for partners, and less uncertainty appraisal for both patients and partners, immediately following the intervention.

#### Coping strategies

Coping strategies were conceptualised as changes in the way patients and partners attempted to cope with the disease as a result of skills learned via the intervention. Of the five studies that assessed coping strategies [4,49,51,52,54], two reported greater improvements (i.e., increased coping efforts [4]; more active engagement with partner [49]) for intervention patients compared to control patients immediately following the intervention. Two studies reported greater improvements for intervention partners compared to control partners immediately following the intervention (i.e., increased coping effort [4,54]). Two studies reported greater improvements for intervention patients compared to control patients, and intervention partners compared to control partners at the final follow-up point (i.e., increased coping effort [4,54]).

#### Self-efficacy

Self-efficacy was conceptualised as patients’ and partners’ perceived competence and confidence in managing stress and assisting with disease related issues (e.g., patient’s symptoms). Of the four studies that assessed self-efficacy [52,54,56,59], one study reported greater improvements for intervention patients compared to control patients immediately following the intervention and at the final follow-up point [54], and another study reported greater improvements for intervention partners compared to control partners immediately following the intervention and at the final follow-up point [59].

#### Couple communication

Couple communication was conceptualised as how couples communicate and discuss thoughts, feelings, and practical issues surrounding the cancer between each other. Of the two studies that assessed communication [4,52], both reported greater improvements for intervention patients and partners than control patients and partners immediately following the intervention with one of these reporting sustained improvements for intervention partners compared to control partners at the final follow-up point [52].

#### Problem solving

Problem solving was conceptualised as how patients and partners approach and manage particular issues and stressors. One study assessed problem-solving and reported greater improvements for intervention patients than control patients immediately following the intervention and at the final follow-up point [50].

## Discussion

The aim of this review was to examine the efficacy, content and delivery of couple-based interventions, and the elements of these interventions that seem most promising in improving adjustment in patients and partners. Most of the couple-based interventions included in this review demonstrated significant improvements for intervention couples compared to control couples, albeit with small to medium effect sizes (*d*~.35-.45), on a range of psychosocial outcomes. The effect sizes reported are similar to those reported in meta-analyses of patient-only and partner/caregiver-only interventions [10,12,13,28]. Couple-based interventions tended to have the greatest impact on improving outcomes such as couple communication, psychological distress, relationship functioning; and in some instances they maintained intervention effects longer than patient-only interventions [4,50]. Two separate meta-analyses have indicated than an important moderator of intervention effects in patient-only interventions is the total time spent with the therapist delivering the intervention [11,12]. Considering the findings of this review and those of these two meta-analyses, it could be suggested that the strengthening of supportive relationships (whether it be the strengthening of the therapeutic relationship or the couple relationship) is key to achieving positive outcomes following psychosocial interventions. Only two studies [4,50] included in this review that compared the efficacy of the couple-based intervention with a comparable patient-only intervention found stronger outcomes for the couple-based intervention at six and 12-month follow-ups compared to the patient-only intervention. Improving support, shared learning, and practicing learned skills may allow easier transfer from the clinical setting to the couple’s natural environment, increasing the likelihood that improvements will be maintained at the conclusion of the intervention [50]. Addressing these skills are even more important when considering population subgroups that may be more at risk for distress.

Some patterns in outcomes emerged when comparing the target population and timing of the intervention, mode of delivery, who delivered the intervention, and specific content of the intervention.

### Target population and timing

Interventions targeting early-stage cancers appeared to result in greater improvements when compared to interventions targeting late-stage or advanced cancers. For instance, improvements in immediate anxiety and QoL were more likely to occur for those with an early-stage diagnosis than for those with a late-stage diagnosis [23,52]. For a late-stage diagnosis, there was evidence to suggest that intervention improved patients’ appraisal of their cancer and feelings of hopelessness, and partners’ appraisal of their caregiving [51,64]. These types of communications perhaps reflect a change in patients’ and partners’ existential position following an arduous cancer journey. Despite couples making improvements in hopelessness and negative illness appraisal from baseline to follow-up, they may still require continued professional support, as the burden of cancer may exceed their resources during these stressful times. It has also been noted that the measurement of mental and physical functioning among such seriously ill populations needs to be more realistic. Rather than measuring at, for example, bi-monthly intervals, the measurement of QoL at more regular intervals (e.g., weekly) and focusing on improving patients’ and partners’ present functioning may be more appropriate than attempting long-term gains [51]. Other interventions targeting self-care and symptom management discussion were found to be efficacious in improving physical distress and both patients’ and partners’ ability to manage symptoms regardless of their diagnosis [48,59].

One study included in this review [53], and a study by Manne and colleagues [3] have concluded that intervention effects were greater for patients with unsupportive partners [3], patients with higher levels of physical distress [3], couples in shorter relationships [53], and patients receiving chemotherapy compared to patients not receiving chemotherapy [53]. This suggests that patients with less supportive partners and couples in shorter relationships may still be developing skills in how to cope with major life stressors together. Similarly, patients that report great physical distress, or are receiving chemotherapy, are potentially facing increased stressors and burdens from their disease. Thus, it may be more appropriate to target particular interventions, or elements of interventions, to specific patient characteristics, to increase the likelihood of a positive outcome for the couple.

### Mode of delivery

No significant differences emerged when comparing face-to-face and telephone delivery of interventions, suggesting that either modality is appropriate for these types of interventions. However, among the studies that included telephone interventions, the majority of participants felt there were benefits being able to talk to a counsellor without leaving their home, though they felt that a degree of face-to-face interaction was still necessary [56].

### Health care professionals delivering the intervention

The vast majority of interventions reviewed were delivered face to face by highly trained health care professionals, including employed psychologists, social workers, or nurses. Most had a masters-level degree, at least, and lengthy experience in the field. Additionally, all studies devised and followed specific intervention protocols and endeavoured to maintain high standards of treatment fidelity through regular reviews of their intervention sessions. However, only 50% of the studies implemented specific training in their interventions, ranging between six and 40 hours.

### Focus of the intervention

Whereas the specific interventions presented in each study were varied in terms of their theoretical framework and delivery, the majority focused on increasing communication between the patient and partner as a means of improving coping and adjustment to cancer. These interventions typically had two broad aims: enhance participants’ ability to express emotion (i.e., uninhibited communication of cancer concerns, and overcoming the propensity of patients and partners to ‘hold back’ concerns) and to communicate needs for managing cancer symptoms (targeting self-care and self-efficacy).

#### Limitations of studies reviewed

The positive outcomes of the couple-based interventions reviewed should be tempered by acknowledgement of some methodological limitations. The methodological strength of the included studies was affected most heavily by a failure to adequately describe attrition rates, randomisation techniques, blinding procedures, as well as limited use of intention-to-treat analyses. There are limitations relating to the cultural generalisability of these findings, given the vast majority of patients participating in these interventions were middle-aged Caucasian women in heterosexual relationships. None of the interventions reviewed here specifically recruited same-sex couples, and only one [56] specifically recruited from a minority population. Campbell et al. [56] recruited African-American prostate cancer patients (a significantly under-represented population) using African-American psychologists, with some success.

### Uptake of couple-based interventions

The utilisation of psychosocial services by patients following a cancer diagnosis is low, with evidence suggesting that less than 20% of patients actively engage with cancer support services [69,70]. Among the couple-based interventions included in this review, approximately 50% of eligible couples agreed to be participate, and were subsequently randomised. Moreover, 74.45% of patients and 71.30% of partners who were randomised provided data at the final follow-up point. Despite these encouraging figures, improving uptake psychosocial interventions for couples should remain a high priority. The most common reasons for refusal of a couple-based intervention were being too busy to participate [22,52,58-60], the intervention not meeting expectations or refusal of group assignment [51,52,58,60], being too ill to participate [23,55,59,60], and living too far away from intervention facilities [47,53,60]. These barriers to the uptake of interventions point to the need for greater flexibility in the content and delivery of psychosocial for patients and their partners. Although telephone interventions were generally well-accepted by participants, some participants indicated they would prefer at least some face-to-face contact; however, face-to-face contact does not appear to be critical for positive outcomes to be achieved with a couple-based intervention. The content of an intervention should vary depending on participants needs when they are invited into the study. Patients, partners, and families often highlight the period immediately following diagnosis as being the most emotionally taxing [71]. Coping-focused interventions may be more beneficial than information-focused interventions during this period. As patients and partners adjust to the shock of a cancer diagnosis, information and symptom-management based interventions may be a simpler and more efficient means of providing support [46]. This is consistent with evidence from a larger study of the utilisation psychosocial services following a cancer diagnosis [69]. Curry and colleagues reported that the most common reasons for refusal of psychosocial services by patients with cancer include services not being suitable at the time they are offered; a preference for self-management; a preference for informal support; and not requiring/wanting any help [69]. Greater flexibility in the way interventions are delivered and greater flexibility in the content of the intervention should lead to improvements in the uptake of psychosocial interventions for couples.

### ‘Usual care’ conditions

Fourteen studies compared a couple-based intervention to a ‘usual care’ condition [47-59,61]. Across these 14 studies, little (if any) detail was provided regarding what the provision of usual care involved. This is similar to the findings of a recent systematic review of best supportive palliative care studies [72]. No reference is made to the clinical guidelines for psychosocial care of patients with cancer as described by the National Comprehensive Cancer Network (NCCN) [73] or the National Health and Medical Research Council (NHMRC) [74], particularly with regard to ongoing screening and follow-up assessment of distressed participants. This lack of methodological rigor may serve to exaggerate the efficacy of couple-based interventions by introducing systematic bias, particularly at larger sites where usual care conditions may vary (e.g., referral to social work team vs. referral to psychologist) [49]. Moreover, comparing couple-based interventions to a usual care conditions does not allow the potential strengths of a couple-based intervention in comparison to patient-only or partner-only intervention to be investigated. Only two studies adequately compared the efficacy of an intervention delivered to an individual (patient-only) compared to the same intervention delivered to a couple, and a usual care group [4,50]. From these studies, the advantages of a couple-based intervention compared to patient or partner-only interventions are much clearer. While both the experimental groups showed greater improvements compared to the usual care group, intervention effects tended to maintained longer among couples than individuals.

#### Limitations of the review

This review did not include studies published in languages other than English or French, conference abstracts, dissertations, or book chapters. Although this ensures that only peer-reviewed studies were included, a publication bias is possible (e.g., ‘file drawer’ problem).

#### Future directions

This review has clarified the current state of the literature on couple-based interventions for patients with cancer and their partners. However, much is still needed in this area. There is a need for large, multi-site, longitudinal RCTs of couple-based interventions. In particular, given the differences in the level of psychological distress reported by men and women [2], more studies are needed to identify what each gender wants or needs from a couple-based intervention. In the context of a couple-based intervention, there is enormous scope to develop content that addresses the needs of the patient and the partner, but also to further develop content that increases understanding of each other’s needs. More intervention studies across a variety of cancer types, particularly among males (e.g., melanoma, testicular cancer), are needed. Available studies for males have primarily focused on men with prostate cancer, which invariably comes with an older sample. More couple-based interventions for younger couples are needed, as it is likely that a much different set of concerns exist for younger couples compared to older couples (e.g., raising a young family, less financial stability, impact on career). Future RCTs should also endeavour to compare target interventions with the same intervention provided to individuals only, and with usual care conditions.

Moreover, whilst delivery of interventions either face-to-face or over the telephone is acceptable, more studies are needed to ascertain couples’ preferences for psychosocial interventions. Recent advancements in technology, in particular the emergence of Smartphone technology and the increase use of online social networking may provide new and exciting opportunities for the delivery of couple-based interventions.

There is also a need for studies that evaluate the relative cost-effectiveness of face-to-face, telephone, computer-based and print-based self-directed (e.g., workbook based interventions [75]) interventions for couples. Of the studies reviewed here, none addressed the costs involved in providing these types of interventions. The related healthcare burden and strain on those delivering interventions must also be investigated. If a couple-based intervention is as efficacious as a patient or partner-only intervention, and the effects are maintained longer, then there may be potential to alleviate some burden on healthcare clinics and professionals.

## Conclusions

In summary, the findings from this review suggest that there are clear benefits to be gained following the implementation of a couple-based intervention. Although more work is needed, there is enough evidence to be confident that these interventions show promising results in reducing distress and improving coping and adjustment to a cancer diagnosis or to cancer symptoms. Collaboration between researchers and clinicians is crucial to ensuring future research builds on this evidence and the development of efficacious, effective, and accessible interventions continues.

## Endnotes

^a^For the purpose of this review, ‘partners’ refers to spousal partners, rather than non-spousal caregivers. This is consistent with the majority of interventions reviewed here, and with widely accepted definitions of dyadic coping [76].

## Competing interests

The authors declare that they have no competing interests.

## Author’s contributions

TR, SL, AG, and BK conceptualised content and direction of systematic review. TR carried out literature search, data extraction, critical review of included studies, collation of data into tables, and was primary author of the manuscript. SL, AG, and BK provided most critical feedback and editing of manuscript, and provided redrafting suggestions and directions for manuscript. TR, SL, and BK reviewed and rated the methodological criteria of all included studies. KK and JT provided expert feedback on structure and content of manuscript, and provided edits and suggestions for redrafting. All authors read and approved the final manuscript.

## Supplementary Material

Additional file 1 **Table S1.** Summary of couple-based interventions. Click here for file
