# Supplementary material for: Do Couple-Based Interventions Make a Difference for Couples Affected by Cancer?: A Systematic Review
Source: BMC Cancer. 2012 Jul 6;12:279. doi: 10.1186/1471-2407-12-279 (PMC3464780; doi:10.1186/1471-2407-12-279)
Supplement: Additional file 1 — Table S1. Summary of couple-based interventions. [file 1471-2407-12-279-S1.doc]

Table S1. Summary of couple-based interventions

| **Author / Design/Methodological Quality/Evidence Level/Intervention Type** | **Description of intervention** |  | **No. Couples** | **Outcome measures at baseline (* indicates patient only; # indicates partner only)** | **Significant outcomes: * <.05; **<.01**  **Cohen’s d (between group comparisons )** |
| --- | --- | --- | --- | --- | --- |
| Badger et al. (2007)/ CCT/Strong/III-1/Dyadic | Three-arm intervention trial  Arm 1: Six, weekly Telephone Interpersonal Counselling intervention (TIP-C) (*M* = 34 minutes) delivered by a psychiatric nurse counsellor (with oncology expertise). No study-specific training.  Arm 2: self-managed, telephone-based exercise intervention (*M* = 11 minutes) not clear who delivered. No study-specific training.  Arm 3: Attention Control (AC) group who received printed information on breast cancer, received follow-up calls (*M* = 7 minutes). No study-specific training. | | *TIP-C* = 38  *Exercise* = 23  *Attention Control =* 37 | Depression: CES-D; Anxiety: PANAS, SF-12, Index of Clinical Stress | Comparisons between TIP-C and AC  6 weeks: **Patients** Anxiety** *d =* 1.31 |
| Badger et al. (2010)/ RCT/Strong/II/Dyadic | Two-arm intervention trial:  Arm 1: Telephone Interpersonal Counselling intervention (TIP-C). 8 weekly phone calls to patient, 4 bi-weekly phone calls to partners (*M* = 31 minutes). Intervention delivered by Masters-level nurse, social worker with psychiatric and oncology expertise. No study-specific training.  Arm 2: Health Education Attention Condition (HEAC). 8 weekly phone calls to patient, 4 bi-weekly phone calls to partners (*M* = 28 minutes). Intervention delivered by research assistants. No study-specific training. | | *TIP-C* = 36  *HEAC* = 35 | Depression: CES-D, Anxiety: PANAS; SF-12; Index of Clinical Stress; Spiritual well-being: subscale of Quality of Life-Breast Cancer; Physical distress: UCLA PCI; MFI; Social well-being scale: PSS-FA | 8 weeks: **Patients** Depression* *d =* .24, Fatigue* *d* = .14, Perceived social support* *d* = .38, Spiritual well-being* *=* .28; **Partners** Depression *d =* .13, Social well-being* *d* = .43, Perceived social support *d* = .32, Spiritual well-being *=* .21  16 weeks: **Patients** Depression *d =* .20, Fatigue *d* = .29, Perceived social support *d* = .38, Spiritual well-being *=* .31; **Partners** Depression *d =* .20, Social well-being *d* = .45, Perceived social support **d* = .48, Spiritual well-being *=* .25 |
| Baucom et al. (2009)/ RCT/Strong/II/Dyadic | Two-arm intervention trial:  Arm 1: Six, face-to-face ,75-minute biweekly Relationship Enhancement (RE) sessions, emphasis on problem-solving skills & emotional expressiveness. Intervention delivered by advanced doctoral students in clinical psychology, trained in couples therapy  Arm 2: Usual care (control condition) | | *RE* = 8  *Control* = 6 | Psychological distress: BSI, PGI; QoL*: Fact-B; SIS; Physical distress: BFI; BPI; RSS; Sexual functioning: DISF; Relationship Functioning: QMI | 12 weeks: **Patients** BSI *d* = .32; PGI *d* = .23; Relationship Functioning *d* = .65; QoL *d* = .38; **Partners** BSI *d* = .16; PGI *d =* .63; Relationship Functioning *d* = .20  12 months: **Patients** BSI *d* = .59; PGI *d* = .12; Relationship Functioning *d* = .11; QoL *d* = .48; **Partners** BSI: *d* = .16; PGI *d* =.49; Relationship Functioning *d* = .25; |
| Budin et al. (2008)/ CCT/Moderate/III-1/Individual | Four-arm intervention trial:  Arm 1: Disease Management (DM; TAU control condition)  Arm 2: DM and standardised psychoeducation (SE); SE delivered by four, disease phase-specific videos  Arm 3: DM and telephone counselling (TC); TC delivered in four disease phase-specific videos by nurses trained in TC methods.  Arm 4: Disease management and standardised psychoeducation and telephone counselling (SE + TC). |  | *SE* = 66  *TC* = 66  *SE + TC* = 58  *Control*  = 59 | Psychological distress: PAIS-SR; PAL-C; Physical distress: SRHS; PAL-C, BCTRI*; Social Adjustment: PAIS | *Means and SDs not available to compute Cohen’s d  T3 (adjuvant therapy)-T4 (ongoing recovery **Patients:** Psychological distress** (TC vs. other groups)  **Partners:** Physical distress* (SE+ TC vs. other groups) |
| Campbell et al. (2006)/ CCT/Moderate/III-1/Dyadic | Two-arm intervention trial:  Arm 1: Six, weekly 60-minute, Coping Skills Training (CST), focused on problem-solving skills, training in cognitive & behavioural coping skills. The session were delivered by a trained, African-American, doctoral level medical psychologist  Arm 2: Usual care (control condition) | | *CST* = 20  *Control* = 20 | QoL*: SF-36; Physical Distress: EPIC, POMS-SF#, CSI# Sexuality: EPIC; Self-efficacy (symptom control): SESCI | 6 weeks: **Patients** Physical Distress* (bowel bother) *d* = .47 |
| Donnelly et al. (2000)/ CCT/Moderate/III-2/Dyadic | Single-arm, pre-post pilot intervention:  Weekly, telephone-based interpersonal psychotherapy (IPT) sessions during chemotherapy delivered by a trained clinical psychologist, undertaking post-doctoral fellowship in psycho-oncology. | | *N* = 13 | Psychological Distress: IES; MHI | n/a |
| Kayser, Feldman, Borstelmann, & Daniels (2010)/ RCT/Moderate/II/Dyadic | Two-arm intervention trial:  Arm 1: Partners in Coping Program (PICP) improve coping ability, supportive communication, assessing couples’ social support, and caring for children, nine 60-minute biweekly face-to-face sessions over an average of 5 months, delivered by a masters-level clinical social worker whom received specific intervention training  Arm 2: Standard Social Work Services (SSWS; control group), couples were provided with the contact details of a social worker who could link them to various services. |  | *PICP* = 36  *Control* = 27 | QoL: FACT-B*, QL-SP#, IIRS# | 6 months: **Patients** Physical well-being *d* = .34; Emotional well-being *d* = .33; Social well-being *d* = .32; Functional well-being *d* = .40; Total FACT-B *d* = .38; **Partners** Emotional well-being *d* = .54; Illness intrusiveness *d* = .38  12 months: **Patients** Physical well-being *d* = .47; Emotional well-being *d* = .55; Social well-being *d* = .27; Functional well-being *d* = .34; Total FACT-B *d* = .44; **Partners** Emotional well-being *d* = .38; Illness intrusiveness *d* = .26 |
| Kozachik et al. (2001)/ CCT/Moderate/III-1/Dyadic | Two-arm intervention trial:  Arm 1: Cancer Care Intervention (CCI), focus on symptom management, emotional support, and caregiver preparedness, coordinating community & family resources; nine contacts (five face-to-face & four via telephone) every two weeks over a course of 16 weeks, delivered by masters-trained oncology nurses.  Arm 2: Usual care (control condition) |  | *Exp* = 61  *Control* = 59 | Depression: CES-D | n/a |
| Kuijer et al. (2004)/ CCT/Moderate/III-1/Dyadic | Two-arm intervention trial:  Arm 1: Focus on improving relationship equity, five 90-minute biweekly face-to-face sessions with a psychologist, no intervention-specific training undertaken.  Arm 2: Wait-list control group |  | *Relationship Equity* = 32  *Control* = 27 | Depression: CES-D; Relationship Functioning: 0-10 Ladder | 10 weeks: **Patients** Depression *d* = 1.1; Relationship Functioning *d* = .63; **Partners** Depression *d* = .02; Relationship Functioning *d* = 1.09  3 months: **Patients** Depression *d* = .45; Relationship Functioning *d* = .19; **Partners** Depression *d* = .10; Relationship Functioning *d* = .18 |
| Kurtz et al. (2005)/ CCT/Moderate/III-1/Coaching | Two-arm intervention trial:  Arm 1: Symptom management intervention, 10 contacts (5 face-to-face, 5 telephone) over 20 weeks with nurse, no intervention specific training undertaken  Arm 2: Treatment as usual (control condition) |  | *Symptom management* = 118  *Control* = 119 | Depression: CES-D; Symptom Severity*/Symptom Assistance#:authors own symptom severity index; Physical/Social Functioning: MOS; SF-36; Self-efficacy#: authors own mastery index | 10 weeks: **Patients** Depression *d* = .23; Symptom Severity: *d =* .28; Physical Functioning: *d* = .18; Social Functioning: *d* = .18; **Partners** Depression *d* = .25; Self-efficacy *d* = .13; Symptom Assistance *d* = .12; Social Functioning *d* = .18  20 weeks: **Patients** Depression *d* = .39; Symptom Severity: *d =* .32; Physical Functioning: *d* = .38; Social Functioning: *d* = .36;  **Partners** Depression *d* = .04; Self-efficacy *d* = .12; Symptom Assistance *d* = .11; Social Functioning *d* = .22 |
| Manne & Badr (2008)/ Cohort/Moderate/III-2/Dyadic | Single-arm intervention trial:  Intimacy-Enhancing Couples’ Therapy (IECT), focus on relationship-enhancing behaviours by improving reciprocal disclosure and responsiveness, viewing the illness in relationship terms; five 1-hour weekly face-to-face sessions with an unspecified therapist trained in specific intervention content |  | *N* = 16 | Psychological Distress: MHI, IES; Relationship functioning: PAIR | ^ Pre-post within group comparison  6 Weeks: **Patients** MHI (Distress)* *d* = .71; IES (Intrusiveness)* *d* = .37; IES (Avoidance)* *d* = .25; PAIR (perceived partner responsiveness)* *d* = .36; PAIR (cancer-specific closeness) *d* = .15; **Partner** MHI (Distress)* *d* = .79; IES (Intrusiveness)* *d* = .96; IES (Avoidance)* *d* = .84 |
| McCorkle et al. (2007)/ CCT/Strong/III-1/Coaching | Two-arm intervention trial:  Arm 1: Standardised Nursing Intervention Protocol (SNIP) for patients and partners following a radical prostatectomy. Focus on monitoring /managing symptoms, teaching self-care, counselling patients and family members;  Arm 2: Usual care (control condition), 16 contacts (8 in-home, 8 telephone) over 8 weeks, delivered by Advanced Practice Nurses and board certified nurse practioners trained in specific intervention content |  | *SNIP* = 62  *Control* = 64 | Depression: CES-D; Sexuality, Relationship Functioning: CARES | 3 months: **Patients** Depression *d* = .23; Relationship Functioning *d* = .10; **Partners** Depression *d* = .11; Relationship Functioning *d* = .41; Sexual Functioning *d* = .27  6 months: **Patients** Depression *d* = .21; Relationship Functioning *d* = .34; Sexual Functioning *d* = .34 **Partners** Depression *d* = .39; Relationship Functioning *d* = .63; Sexual Functioning *d* = .51 |
| McLean et al. (2008)/ Cohort/Moderate/III-2/Dyadic | Single-arm intervention trial:  Adaptation of Emotionally Focused Couple Therapy (EFT) emphasises the sharing of emotions, normalizing feelings of separation, 8-20 face-to-face sessions with doctoral-level psychologists with some training in EFT |  | *N* = 16 | Depression: BDI-II, BHS; Relationship Functioning: RDAS | ^Comparisons to baseline scores  After eight sessions: **Patients** BDI-II *d* = .33; BHS *d* = .23; RDAS *d* = 1.07; **Partners** BHS *d* = .16; RDAS *d* = .67  3 months: : **Patients** BDI-II *d* = .54; BHS *d* = .17; Relationship Functioning *d* = .84; **Partners** BDI-II *d* = .29; BHS *d* = .18; Relationship Functioning *d* = .76; |
| McLean et al. (2011)/ RCT/Strong/II/Dyadic | Two-arm intervention trial:  Arm 1: Adaptation of Emotionally Focused Couple Therapy (EFT) emphasises the sharing of emotions, normalizing feelings of separation; eight, one-hour weekly face-to-face sessions with psychologist trained in EFT  Arm 2: Usual care (control group) |  | *EFT* = 22  *Control* = 20 | Depression: BDI-II, BHS; CBS-T#; CBS-D# Relationship Functioning: RDAS; Coping: RFCS* | 8 weeks: **Patients** Relationship Functioning** *d* = 1.65; Coping* *d* = .36; **Partners** Relationship Functioning** *d* = 2.03  12 weeks: **Patients** Relationship Functioning** *d* = 1.32; Coping* *d* = .37; **Partners** Relationship Functioning** *d* = 1.22 |
| Mohr et al. (2003)/ Cohort/Moderate/III-2/Dyadic | Single-arm intervention trial:  Focus on facilitating shifts in beliefs, goals and values, facilitating conversations about death and dying, increasing intimacy and emotional support; eight one-hour weekly face-to-face sessions with PhD-level psychologist or clinical social worker, no intervention-specific training undertaken |  | *N =* 6 | Anxiety: authors own measure (distress & worry about dying); Depression: BDI-II; QoL: SF-36; Relationship Functioning: authors own positive/negative scale; Social Support: PSSS* (positive & negative); ZCBZ# | ^ Pre-post within group comparison  Eight weeks: **Patients** Anxiety (distress about dying) *d* = .54; Relationship functioning (positive) *d* = .74; **Partner** Anxiety (worry about dying) *d* = .38 |
| Nezu et al. (2003)/ RCT/Strong/II/Coaching | Three-arm intervention trial:  Arm 1: Problem-solving therapy for patients and a supportive other (PST-SO), focus on enhancing problem-solving skills, ten 90-minute weekly face-to-face sessions, masters level psychologists, social workers, and nurses, t15 hours training in intervention-specific content  Arm 2: Problem-solving therapy for patients only (PST)  Arm 3: Wait-list control group. |  | *PST-SO* = 50  *PST* = 50  *Control* = 50 | QoL*: CR, CARES; Psychological Distress*: CR(HRSD), POMS, BSI, KAS-R#; Problem Solving: SPSI-R | ^Comparisons between PST-SO and PST  10 weeks: **Patients** POMS *d* = .18; BSI *d* = .33; CARES *d* = .26; Problem Solving *d* = .08; **Partners** KASR-(Psychiatric) *d* = .13; KASR-(Social) *d* = .16  12 months: **Patients** POMS *d* = .44; BSI *d* = .84; CARES *d* = .67; Problem Solving *d* = .57; **Partners** KASR-(Psychiatric) *d* = .99; KASR (Social) *d* = .31 |
| Northouse et al. (2005)/ CCT/Strong/III-1/Dyadic | Two-arm intervention:  Arm 1: FOCUS program (interventions: family involvement, optimistic attitude, coping effectiveness, uncertainty reduction and symptom management; initial intervention phase: three 90-minute home visits spaced one-month apart/Booster phase: Two phone calls to both patient and caregiver (~30 minutes/call) provided after 3-month follow-up, delivered by masters-level nurse, trained in the FOCUS program  Arm 2: Usual care (control condition) |  | *N* = 182 | QoL & Physical Distress: FACT-B, FACT-G, SF-36; Appraisal: AIS, MUIS, BHS; Coping: BriefCOPE | 3 months: **Patients** AIS *d* = .04; BHS *d* = .10; **Partners** AIS* *d* = .19  6 months: **Patients** AIS* *d* = .13; BHS *d* = .17; **Partners** AIS *d* = .05 |
| Northouse et al. (2007)/ CCT/Strong/III-1/Dyadic | Two-arm intervention:  Arm 1: FOCUS program (intervention): family involvement, optimistic attitude, coping effectiveness, uncertainty reduction and symptom management; three 90-minute home visits spaced and two phone calls to both patient and caregiver (~30 minutes/call) spaced two-weeks apart, between baseline and 4-month follow-up, delivered by masters-level nurse, trained in the FOCUS program  Arm 2: Usual care (control condition) |  | *Focus* = 129  *Control* = 134 | QoL & Physical Distress: FACT-G, SF-36, EPIC; Sexuality: EPIC; Appraisal: MUIS, BHS, AIS; Coping: BriefCOPE; Self-Efficacy*: LCSES; Communication: LMISS | 4-months: **Patients** MUIS = .22; LMISS *=* .22; **Partners** SF-36M/FACT-G = .25, .26; Appraisal (all): = .27/.32; LCSES = .26; LMISS = .31, EPIC (urinary, symptom distress) = .30, .34  12 months: **Partners** LCSES = .27, BriefCOPE (active coping) = .28, LMISS = .29, SF-36P = .32 |
| Northouse et al. (2011)/ RCT/Moderate/II/Dyadic | Three-arm intervention:  Arm 1: Brief FOCUS program, family involvement, optimistic attitude, coping effectiveness, uncertainty reduction and symptom management; 2 x 90-minute home visits, one 30 minute phone call, delivered by masters-level nurse trained in the FOCUS program  Arm 2: Extensive FOCUS program, family involvement, optimistic attitude, coping effectiveness, uncertainty reduction and symptom management; 4 x 90-minute home visits, two 30 minute phone calls, delivered by masters-level nurse trained in the FOCUS program  Arm 3: Usual care (control condition) |  | *Brief* = 159  *Extensive*  = 162  *Control* = 163 | QoL: FACT-G, SF-36; Psychological Distress: RFD; Appraisal: AIS, MUIS, BHS; Coping: BriefCOPE; Healthy Lifestyle: author’s scale; Self-efficacy: author’s scale; Dyadic support: SSQ | ^ Comparisons between Brief and Extensive  3 months: **Patients** Self-efficacy* *d* = .15; **Partners** Coping (Avoidant)* *d* = .41; Dyadic support* *d* = .27; QoL* *d* = .25  6 months: **Patients** Self-efficacy* *d* = .24; **Partners** Dyadic support* d = .10; Healthy lifestyle* *d* = .15 |
| Porter et al. (2009)/ CCT/Moderate/III-1/Dyadic | Two-arm intervention:  Arm 1:Partner-assisted emotional disclosure (PAED) focused on decreasing the ‘holding back’ of cancer-related disclosures to partners, increasing relationship quality and intimacy, decreasing psychological distress; 4 face-to-face sessions with masters-level social worker or psychologist  Arm 2: Partner-assisted educational (EDU) intervention received general cancer information only |  | *PAED* = 65  *EDU* = 65 | Psychological Distress: POMS-SF; Relationship Functioning: QMI; MSIS | *Means and SDs not available to compute Cohen’s d  Post-intervention **Patients:** QMI**; MSIS* |
| Scott, Halford, & Ward (2004)/ CCT/Moderate/III-1/Dyadic | Three-arm intervention:  Arm 1: MI intervention: educational materials regarding patients’ particular cancer and associated treatments, no specific psychological intervention provided, five 15-minute phone calls, medical information booklets  Arm 2: PC intervention: combined MI intervention with supportive counselling and education in coping skills, four 2-hour sessions: pre & post-surgery, 1 week & 6-months after; two 30-minute phone calls 1 & 3-months post-surgery delivered face-to-face by three female psychologists, with 3-15 years experience.  Arm 3: CanCOPE: same as PC intervention, but undertaken with couples focus on teaching of supportive communication skills |  | *N* = 94 | Psychological Distress: IES; Sexuality: PAIS-SR SSS, BISF; Coping: WOC-CA; Communication: qualitative interviews | ^ Comparisons between CanCOPE and PC  Post-intervention **Patients** Coping** *d* = .25; IES (Avoidance)* *d* = .36; SSS *d* = .56; BISF (Desire) *d* = .50; **Partners** Coping* *d* = .36  12 months **Patients** Coping* *d* = .82; IES (Avoidance)* *d* = .55; SSS *d* = .39; BISF (desire) *d* = .08; **Partners** Coping* *d* = .61 |
| Shields & Rousseau (2004)/ Cohort/Moderate/III-2/Dyadic | Three-arm intervention trial:  Arm 1: 2-session workshop intervention , focus on comparing, contrasting patient/partner experiences of cancer, increasing communication, helping couples find meaning; delivered face-to-face by unspecified therapist  Arm 2: 1-session workshop intervention (as above)  Arm 3: No treatment control group. |  | *2-sessions* = 12  *1-sessions* = 21  *Control* = 15 | Psychological distress: SF-12; IES; Relationship Functioning: RDAS | n/a |
| Thornton, Perez, & Meyerowitz (2004)/ CCT/Moderate/III-1/Dyadic | Two-arm intervention:  Arm 1: Brief (45 minute) one-off supportive intervention pre-surgery focusing on improving communication within couple and between couple and medical team, delivered face-to-face by unspecified counsellor  Arm 2: Usual care (control group) |  | *N=65* | QoL: MOS, FACT-P; Physical Distress: UIS; Psychological Distress: PANAS, IES, PSS-FA; Relationship Functioning: RDAS | 3 weeks: **Patients** FACT-P (social/family well-being)* *d* = .58; **Partners** PSS-FA* *d* = .29;  12 months: **Partners** PSS-FA**d* = .54 |

Centre for Epidemiological Studies - Depression Scale (CES-D); Positive and Negative Affect Schedule (PANAS;); SF-12 (anxiety measure); Caregiver Strain Index (CSI); Index of Clinical Stress; UCLA Prostate Cancer Index (UCLA PCI); Multidimensional Fatigue Inventory (MFI); Perceived Social Support-Family scale (PSS-FA); Brief Symptom Inventory (BSI; psychological distress measure); Posttraumatic Growth Inventory (PGI); Functional Assessment of Cancer Therapy-Breast (FACT-B-QoL measure); Functional Assessment of Cancer Therapy-General (FACT-G, QoL measure); Self-image scale (SIS – qol measure); Brief Fatigue Inventory (BFI); Brief Pain Inventory (BPI); Quality of Life Questionnaire for Spouses (QL-SP); Illness intrusiveness Rating Scale (IIRS); Rotterdam Symptom Scale (RSS; physical distress); Derogatis Inventory of Sexual Functioning (DISF); Psychosocial Adjustment to Illness Scale - Self Report (PAIS-SR); Profile of Adaptation to Life Clinical Scale -Psychological Well-being subscale (PAL-C); Self-rated Health Subscale (SRHS; physical distress); PAL-C - physical symptoms subscale; Breast Cancer Treatment Response Inventory (BCTRI); BCTRI Side Effects Severity subscale; PAIS- Vocational, Domestic, Social subscale (social adjustment measure); Short-Form Health Survey (SF-36; QoL measure); Expanded Prostate Cancer Index Composite (EPIC; physical distress and sexuality measure); Self-Efficacy for Symptom Control Inventory (SESCI); Impact of Event Scale (IES); Mental Health Inventory (MHI); Medical Outcomes Study (MOS); Cancer Rehabilitation Evaluation System (CARES); Partner Unsupportive Behaviours Scale (PUBS, from Partner Responses to Cancer Inventory); Beck Depression Inventory (BDI-II); Beck Hopelessness Scale (BHS); Revised Dyadic Adjustment Scale (RDAS); *CR – Clinican Rated*; *Hamilton Rating Scale for Depression*; Profile of Mood States (POMS); Katz Adjustment Scale-Relative’s Form (KAS-R; *partner rated*); Relationship-Focused Coping Scale (RFCS); Mishel Uncertainty in Illness Scale (MUIS); Appraisal of Illness Scale (AIS) ; Brief Coping Orientations to Problems Experienced (Brief-COPE); Lewis Cancer Self-Efficacy Scale (LCSES); Lewis Mutuality and Interpersonal Sensitivity Scale (LMISS); Risk of Distress Scale (RFD); Quality of Marriage Index (QMI) ; Miller Social Intimacy Scale (MSIS); Profile of Mood States- Short Form (POMS-SF); Sexual Self Schema (SSS) for Women; Brief Index of Sexual Functioning (BISF); Revised Ways of Coping Questionnaire - Cancer Version (WOC-CA); Caregiver Burden Scale (Time subscale); Caregiver Burden Subscale (Difficulty subscale); Dyadic support: Social Support Questionnaire; Personal Assessment of Intimacy in Relationships inventory (PAIR), Functional Assessment of Cancer (Prostate), Urinary Incontinence Scale (UIS)
